# An introduction to data reduction: space-group determination, scaling and intensity statistics

**DOI:** 10.1107/S090744491003982X

**Published:** 2011-03-18

**Authors:** Philip R. Evans

**Affiliations:** a MRC Laboratory of Molecular Biology, Hills Road, Cambridge CB2 0QH, England

**Keywords:** *CCP*4, data reduction, data scaling, software

## Abstract

This paper presents an overview of how to run the *CCP*4 programs for data reduction (*SCALA*, *POINTLESS* and *CTRUNCATE*) through the *CCP*4 graphical interface *ccp*4*i* and points out some issues that need to be considered, together with a few examples. It covers determination of the point-group symmetry of the diffraction data (the Laue group), which is required for the subsequent scaling step, examination of systematic absences, which in many cases will allow inference of the space group, putting multiple data sets on a common indexing system when there are alternatives, the scaling step itself, which produces a large set of data-quality indicators, estimation of |*F*| from intensity and finally examination of intensity statistics to detect crystal pathologies such as twinning. An appendix outlines the scoring schemes used by the program *POINTLESS* to assign probabilities to possible Laue and space groups.

## Abstract 2

A summary of how to run the data-reduction programs in the *CCP*4 suite.

## 1. Introduction

Estimates of integrated intensities from X-ray diffraction images are not generally suitable for immediate use in structure determination. Theoretically, the measured intensity *I*_**h**_ of a reflection **h** is proportional to the square of the underlying structure factor |**F**_**h**_|^2^, which is the quantity that we want, with an associated measurement error, but systematic effects of the diffraction experiment break this proportionality. Such systematic effects include changes in the beam intensity, changes in the exposed volume of the crystal, radiation damage, bad areas of the detector and physical obstruction of the detector (*e.g.* by the backstop or cryostream). If data from different crystals (or different sweeps of the same crystal) are being merged, corrections must also be applied for changes in exposure time and rotation rate. In order to infer |**F**_**h**_|^2^ from *I*_**h**_, we need to put the measured intensities on the same scale by modelling the experiment and inverting its effects. This is generally performed in a scaling process that makes the data internally consistent by adjusting the scaling model to minimize the difference between symmetry-related observations. This process requires us to know the point-group symmetry of the diffraction pattern, so we need to determine this symmetry prior to scaling. The scaling process produces an estimate of the intensity of each unique reflection by averaging over all of the corrected intensities, together with an estimate of its error σ(*I*_**h**_). The final stage in data reduction is estimation of the structure amplitude |**F**_**h**_| from the intensity, which is approximately *I*_**h**_^1/2^ (but with a skewing factor for intensities that are below or close to background noise, *e.g.* ‘negative’ intensities); at the same time, the intensity statistics can be examined to detect pathologies such as twinning.

This paper presents a brief overview of how to run *CCP*4 programs for data reduction through the *CCP*4 graphical interface *ccp*4*i* and points out some issues that need to be considered. No attempt is made to be comprehensive nor to provide full references for everything. Automated pipelines such as *xia*2 (Winter, 2010 ▶) are often useful and generally work well, but sometimes in difficult cases finer control is needed. In the current version of *ccp*4*i* (*CCP*4 release 6.1.3) the ‘Data Reduction’ module contains two major relevant tasks: ‘Find or Match Laue Group’, which determines the crystal symmetry, and ‘Scale and Merge Intensities’, which outputs a file containing averaged structure amplitudes. Future GUI versions may combine these steps into a simplified interface. Much of the advice given here is also present in the *CCP*4 wiki (http://www.ccp4wiki.org/).

## 2. Space-group determination

The true space group is only a hypo­thesis until the structure has been solved, since it can be hard to distinguish between exact crystallographic symmetry and approximate noncrystallographic symmetry. However, it is useful to find the likely symmetry early on in the structure-determination pipeline, since it is required for scaling and indeed may affect the data-collection strategy. The program *POINTLESS* (Evans, 2006 ▶) examines the symmetry of the diffraction pattern and scores the possible crystallographic symmetry. Indexing in the integration program (*e.g. MOSFLM*) only indicates the lattice symmetry, *i.e.* the geometry of the lattice giving constraints on the cell dimensions (*e.g.* α = β = γ = 90° for an orthorhombic lattice), but such relationships can arise accidentally and may not reflect the true symmetry. For example, a primitive hexagonal lattice may belong to point groups 3, 321, 312, 6, 622 or indeed lower symmetry (*C*222, 2 or 1). A rotational axis of symmetry produces identical true intensities for reflections related by that axis, so examination of the observed symmetry in the diffraction pattern allows us to determine the likely point group and hence the Laue group (a point group with added Friedel symmetry) and the Patterson group (with any lattice centring): note that the Patterson group is labelled ‘Laue group’ in the output from *POINTLESS*. Translational symmetry operators that define the space group (*e.g.* the distinction between a pure dyad and a screw dyad) are only visible in the observed diffraction pattern as systematic absences, along the principal axes for screws, and these are less reliable indicators since there are relatively few axial reflections in a full three-dimensional data set and some of these may be unrecorded.

The protocol for determination of space group in *POINTLESS* is as follows.

(i) From the unit-cell dimensions and lattice centring, find the highest compatible lattice symmetry within some tolerance, ignoring any input symmetry information.
(ii) Score each potential rotational symmetry element belonging to the lattice symmetry using all pairs of observations related by that element.
(iii) Score combinations of symmetry elements for all possible subgroups of the lattice-symmetry group (Laue or Patterson groups).
(iv) Score possible space groups from axial systematic absences (the space group is not needed for scaling but is required later for structure solution).
(v) Scores for rotational symmetry operations are based on correlation coefficients rather than *R* factors, since they are less dependent on the unknown scales. A probability is estimated from the correlation coefficient, using equivalent-size samples of unrelated observations to estimate the width of the probability distribution (see Appendix *A* [App appa]).

### 2.1. A simple example

**Figure 1:**
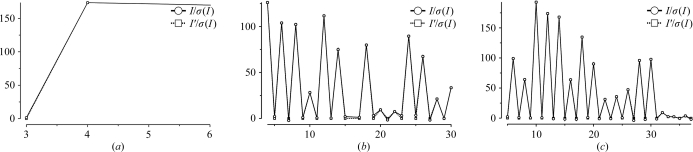
Plots from *POINTLESS* of axial reflections for the *P*2_1_2_1_2_1_ example shown in Table 1 ▶: (*a*) *h*00, (*b*) 0*k*0, (*c*) 00*l*. In each case *I*/σ(*I*) alternates between weak and strong for odd and even indices, respectively, indicating a 2_1_ screw axis in each direction. With only three observations along the *h*00 axis, assignment of a screw along **a** is far less certain than along **b** and **c** (see Table 1 ▶ *c*). The plot of *I*′/σ(*I*) (almost the same in this case) uses a modified value of *I*, subtracting 2% of the neighbouring axial reflection to allow for possible contamination of weak reflections by a strong neighbour. All panels in Figs. 1–5 are monochrome versions of plots from *LOGGRAPH* essentially as they appear from *ccp*4*i*.

*POINTLESS* may be run from the ‘Data Reduction’ module of *ccp*4*i* with the task ‘Find or Match Laue Group’ or from the ‘QuickSymm’ option of the *iMOSFLM* interface (Battye *et al.*, 2011 ▶). Unless the space group is known from previous crystals, the appropriate major option is ‘Determine Laue group’. To use this, fill in the boxes for the title, the input and output file names and the project, crystal and data-set names (if not already set in *MOSFLM*). Table 1 ▶ shows the results for a straightforward example in space group *P*2_1_2_1_2_1_. Table 1 ▶(*a*) shows the scores for the three possible dyad axes in the orthorhombic lattice, all of which are clearly present. Combining these (Table 1 ▶ *b*) shows that the Laue group is *mmm* with a primitive lattice, Patterson group *Pmmm*. Fourier analysis of systematic absences along the three principal axes shows that all three have alternating strong (even) and weak (odd) intensities (Fig. 1 ▶ and Table 1 ▶ *c*), so are likely to be screw axes, implying that the space group is *P*2_1_2_1_2_1_. However, there are only three *h*00 reflections recorded along the a* axis, so confidence in the space-group assignment is not as high as the confidence in the Laue-group assignment (Table 1 ▶ *d*). With so few observations along this axis, it is impossible to be confident that *P*2_1_2_1_2_1_ is the true space group rather than *P*22_1_2_1_.

### 2.2. A pseudo-cubic example

Table 2 ▶ shows the scores for individual symmetry elements for a pseudo-cubic case with *a* ≃ *b* ≃ *c*. It is clear that only the orthorhombic symmetry elements are present: these are the high-scoring elements marked ‘***’. Neither the fourfolds characteristic of tetragonal groups nor the body-diagonal threefolds (along 111 *etc*.) characteristic of cubic groups are present. The joint probability score for the Laue group *Pmmm* is 0.989. The suggested solution (not shown) interchanges *k* and *l* to make *a* < *b* < *c*, which is the IUCr standard convention for a primitive ortho­rhombic cell (Mighell, 2002 ▶). Scoring the possible symmetry elements separately may allow the program and the user to distinguish between true crystallographic symmetry and pseudo-symmetry (*i.e.* a noncrystallographic rotation close to a potential crystallographic rotation), although either the program or the user may be fooled by twinning or if the pseudo-symmetry is very close to crystallo­graphic. If the data were integrated with cell constraints from a higher symmetry than is present, integration should be repeated with the looser cell constraints for the correct symmetry class.

### 2.3. Alternative indexing

If the true point group is lower symmetry than the lattice group, alternative valid but non-equivalent indexing schemes are possible related by symmetry operators that are present in the lattice group but not in the point group (note that these are also the cases in which merohedral twinning is possible). For example, in space group *P*3 (or *P*3_1_) there are four different schemes: (*h*, *k*, *l*), (−*h*, −*k*, *l*), (*k*, *h*, −*l*) or (−*k*, −*h*, −*l*). Alternate indexing ambiguities may also arise from special relationships between unit-cell parameters (*e.g. a* = *b* in an orthorhombic system). For the first crystal (or part data set) any indexing scheme may be chosen, but for subsequent ones autoindexing will randomly pick one setting which may be inconsistent with the original choice. *POINTLESS* can compare a new test data set with a previously processed reference data set (from a merged or unmerged file) and choose the most consistent option (option ‘Match index to reference’ in *ccp*4*i*). In this option, the space group in the reference file is assumed to be correct.

### 2.4. Combining multiple files and multiple wavelengths

Multiple files, *e.g.* from multiple runs of *MOSFLM*, can be combined in *POINTLESS* using the ‘Add file’ button in *ccp*4*i*. They may be combined into a single data set with the same Project, Crystal and Dataset names (button ‘Assign to the same data set as the previous file’) or assigned to different data sets in the case of multiple-wavelength data. Note that the data-set name is used in downstream programs to label columns in the MTZ file, so should be short. Batch numbers are automatically incremented by a multiple of 1000 if necessary to make them unique across all files. If alternative indexing schemes are possible in the lattice group determined from the cell dimensions, then second and subsequent files are compared with the previous ones in the same way as if a reference file were given. Note that if the Laue group symmetry of the first file is wrong this may lead to wrong answers in some cases, so there is an option to determine the Laue symmetry of the first file before reading the rest.

## 3. Scaling

Scaling tries to make symmetry-related and duplicate measurements of a reflection equal by modelling the diffraction experiment, principally as a function of the incident and diffracted beam directions in the crystal (Hamilton *et al.*, 1965 ▶; Fox & Holmes, 1966 ▶; Kabsch, 1988 ▶, 2010 ▶; Otwinowski *et al.*, 2003 ▶; Evans, 2006 ▶). This makes the data internally consistent, assuming that the correct Laue group has been determined. After scaling, the remaining differences between observations can be analysed to give an indication of data quality, though not necessarily of its absolute correctness. In the *ccp*4*i* interface, the task ‘Scale and Merge Intensities’ runs *SCALA* to scale and merge the multiple observations of the same unique reflection, followed by *CTRUNCATE* to infer |*F*| from the intensity *I* and optionally generate or copy a test set of reflections for *R*_free_. The input file may be the output of *POINTLESS*. The *ccp*4*i* task presents a large number of options, but in most cases the defaults are suitable. If you know that you have a significant anomalous scatterer in the crystal, the the option to ‘Separate anomalous pairs for merging statistics’ should be selected, since this allows for real differences between Bijvoet-related reflections *hkl* and *−h −k −l* (very small anomalous differences are probably treated better without this option). Other useful options, after the first run, include setting the high-resolution limit (after deciding on the ‘true’ resolution, see below) and excluding some batches or batch ranges (in the ‘Excluded Data’ tab).

### 3.1. Measures of internal consistency

**Figure 2:**
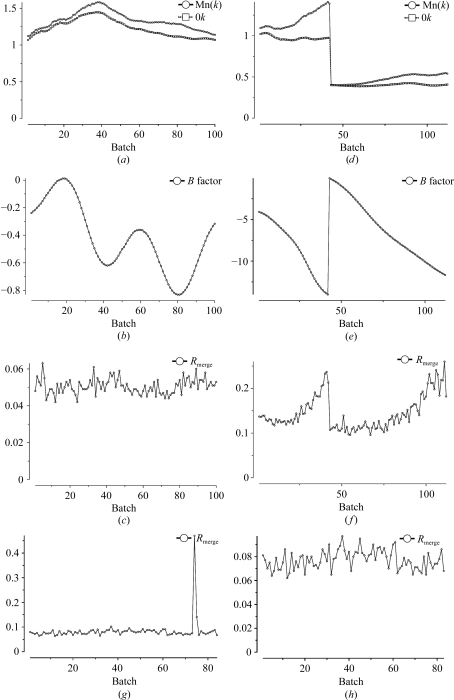
Plots from *SCALA* against ‘batch’ (image) number (*a*–*c*) for a good case with little radiation damage (see text) and (*d*–*f*) for a case with two crystals both suffering radiation damage. (*a*, *d*) Mean scale [Mn(*k*)] and scale at θ = 0° (0*k*); these diverge if the relative *B* factor is large. (*b*, *e*) Relative *B* factor in the scaling; a large and declining negative value (*e*) indicates progressive radiation damage. (*c*, *f*) *R*_merge_ is roughly constant in the good case (*c*) but increases with radiation damage (*f*). (*g*) A plot of *R*_merge_ against batch shows a single outlier arising from a weak or blank image: omitting this batch (*h*) removes this problem.

The traditional measure of internal consistency is *R*_merge_ (also known as *R*_sym_), which is defined as 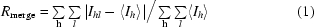
(*i.e.* summed over all observations *l* of reflection **h**), but this has the disadvantage that it increases with the data multiplicity, even though the merged data are improved by averaging more observations. An improvement is the multiplicity-weighted *R*_meas_ or *R*_r.i.m._ (Diederichs & Karplus, 1997 ▶; Weiss & Hilgenfeld, 1997 ▶; Weiss, 2001 ▶), which is defined as
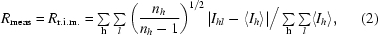
where *n*_*h*_ is the number of observations of reflection **h** [note that in Evans (2006 ▶) the square-root was incorrectly omitted]. A related measure is the precision-indicating *R* factor, which estimates the data quality after merging, 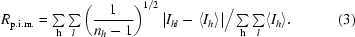
After scaling, *SCALA* outputs a large number of statistics, mostly presented as graphs, and a final summary table which contains most of the data required for the traditional ‘Table 1’ (or perhaps Table S1) in a structural paper. Analyses against ‘batch number’, *i.e.* image number or time, are useful to check for the effects of radiation damage and for bad batches (*e.g.* blank images) or bad regions (Fig. 2 ▶). Individual blank or bad images can be rejected in *SCALA* (see Figs. 2 ▶ *g* and 2 ▶ *h*), but if there are bad regions it may be best to check the integration process carefully. Decisions on where to cut back data to a point where radiation damage is tolerable, or how best to combine data from different crystals or sweeps, are more complicated and tools to explore the best compromise between damage and completeness are not yet well developed, although the program *CHEF* (Winter, 2009 ▶) used in *xia*2 provides a guide.

**Figure 3:**
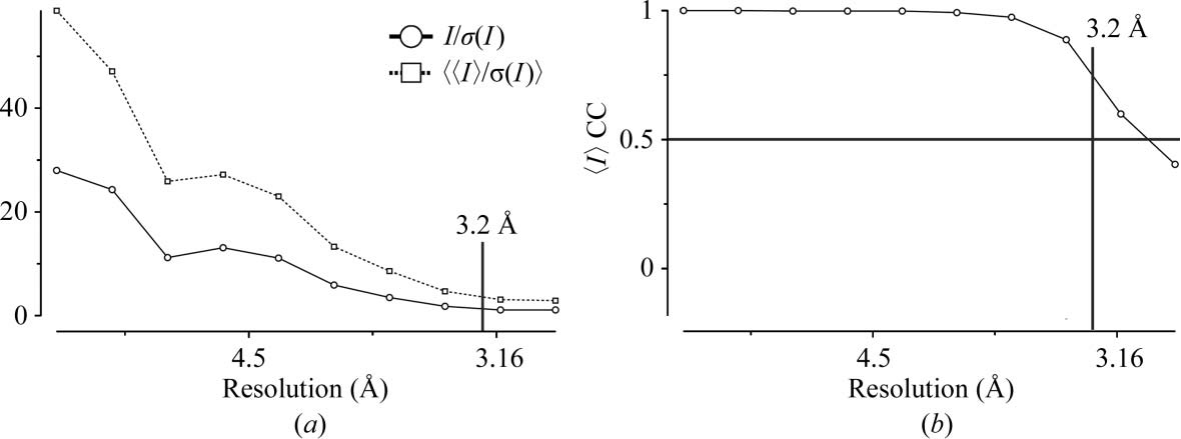
Plots from *SCALA* against resolution. A suitable resolution cutoff may be estimated from a plot of 〈〈*I*〉/σ(*I*)〉, *i.e.* after averaging, where it falls below ∼2 or flattens out [top line in (*a*)] or from the correlation coefficient between 〈*I*〉 for random halves of the observations.

Analyses against resolution suggest whether a resolution cutoff should be applied. The decision on the ‘real’ resolution is not easy: ideally, we would determine the point at which adding the next shell of data is not adding any statistically significant information. The best cutoff point may depend on what the data are to be used for: experimental phasing techniques work on amplitude differences, which are less accurate than the amplitudes themselves. Useful guidelines are the point at which 〈〈*I*_*h*_〉/σ(〈*I*_*h*_〉)〉 [after merging and adjusting the σ(*I*) estimates] falls below about 2, where 〈*I*_*hl*_/σ(*I*_*hl*_)〉 (before merging) falls below about 1, where the correlation coefficient between random half-data-set estimates of 〈*I*_*h*_〉 falls below about 0.5 or where 〈*I*〉 flattens out with respect to resolution; *R*_merge_ is not a very useful criterion. Fig. 3 ▶ shows an example in which the cutoff was set to 3.2 Å using a combination of these criteria. If the data are severely anisotropic then these limits may be relaxed to keep useful data in the best direction.

Analyses of consistency against intensity are not generally useful, since the statistics will always be worse for weak data; however, *R*_merge_ in the top intensity bin should be small. Analysis against intensity is useful in improving estimates of σ(*I*); see Appendix *B* [App appb].

### 3.2. Completeness

**Figure 4:**
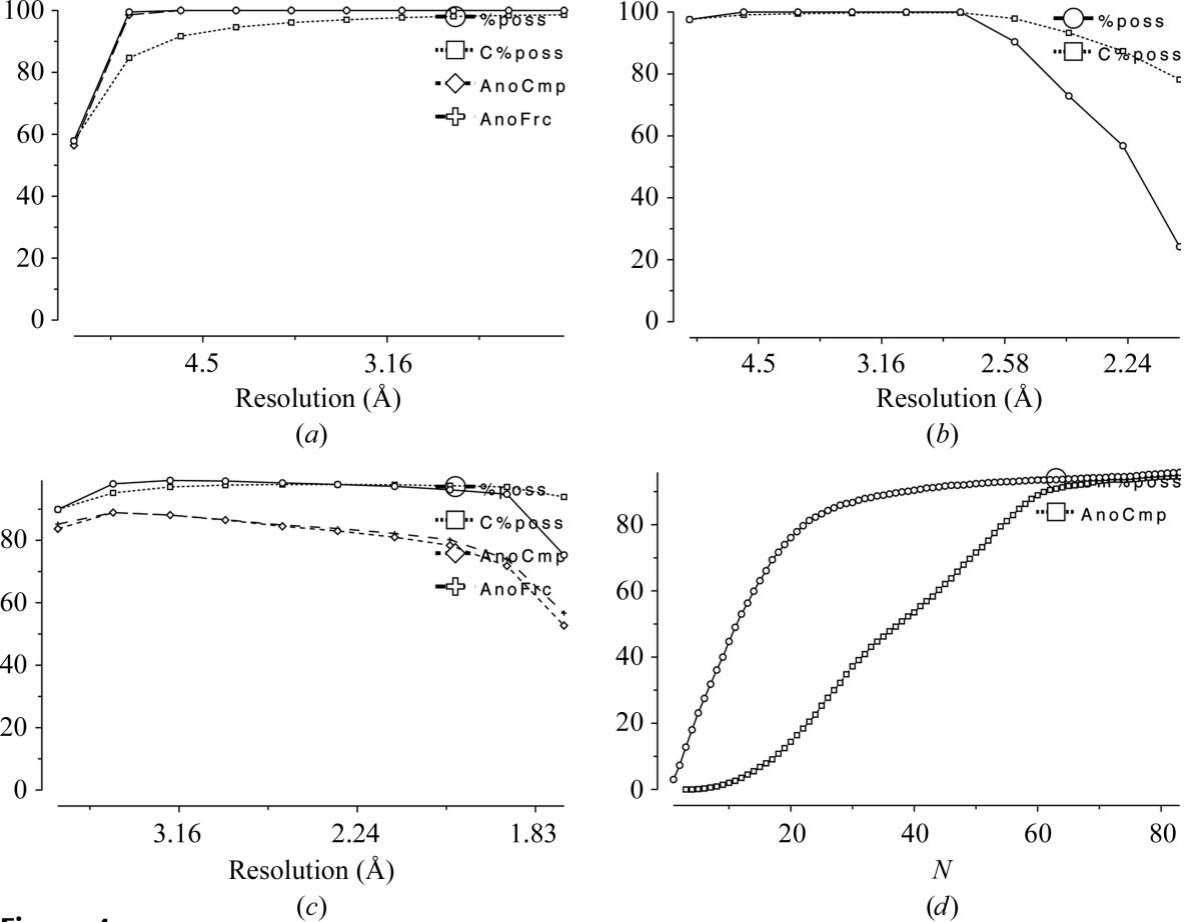
Plots of data completeness against resolution and batch. (*a*) Incompleteness at low resolution owing to detector overloads. (*b*) Incompleteness at high resolution owing to integrating into the corners of a square detector. (*c*) Incompleteness of anomalous data. (*d*) Cumulative completeness against batch (plot not yet available in *SCALA*).

Data completeness is important, preferably in all resolution shells, although it may be less important at the outer edge. James Holton (Advanced Light Source, Lawrence Berkeley National Laboratory, Berkeley, California, USA) has produced a series of instructive movies (http://ucxray.berkeley.edu/~jamesh/movies/) showing the degradation of map quality with systematic incompleteness, such as missing a wedge of data from an incomplete rotation range or losing the strongest reflections as detector overloads: random incompleteness (*e.g.* from omitting an *R*_free_ test set), on the other hand, has little effect on maps. The data-collection strategy should always aim to collect a complete set of data. Plots against resolution from *SCALA* may show incompleteness at low resolution owing to detector overloads (Fig. 4 ▶ *a*), at high resolution owing to integrating into the corners of a square detector (Fig. 4 ▶ *b*) or incompleteness of the anomalous data (Fig. 4 ▶ *c*) which will limit the quality of experimental phasing. Fig. 4 ▶(*d*) shows a plot of cumulative completeness against batch number in an 84° sweep: note that 100% completeness is not reached until the end and that the anomalous completeness lags behind the total completeness by an amount that depends on the symmetry. This plot is not yet implemented in *SCALA*, but when it is it may help in judging the trade-off between completeness and radiation damage.

### 3.3. Outliers

Most data sets contain a small proportion of measurements that are just ‘wrong’ (from which no useful information about the true intensity can be extracted). These arise from various causes, notably diffraction from ice crystals or superfluous protein crystal lattices (crystal clusters) that superimposes on a few (or, in bad cases, many) of the reflections from the crystal of interest. Detection of these intensity outliers is reasonably reliable if the multiplicity is high, but is not possible if there are only one or two observations (if two disagree, which one is correct?). This is a good reason for collecting high-multiplicity data. If *SCALA* is told that there are anomalous differences then the outlier check for discrepancies between Bijvoet-related reflections *I* ^+^ and *I* ^−^ uses a larger tolerance than that used within the *I* ^+^ or *I* ^−^ sets, depending (rather crudely) on the average size of the anomalous differences. The outlier-rejection algorithm assumes that the majority of symmetry-related observations of a reflection are correct: this may fail for reflections behind the backstop, so it is important that the backstop shadow should be identified properly in *MOSFLM*. *SCALA* produces a plot of outliers in their position on the detector (ROGUEPLOT file), which may show outliers clustered around the ice rings or around the backstop, in which case these regions of the detector should be masked out in *MOSFLM*. There is also a list of outliers in the ROGUES file which may be useful to understand the rejects. The rejection limits are set as multiples of the standard deviations and can be altered by the user. When trying to use a weak anomalous signal it may be useful to reduce the limits and eliminate more outliers.

## 4. Detecting anomalous signals

**Figure 5:**
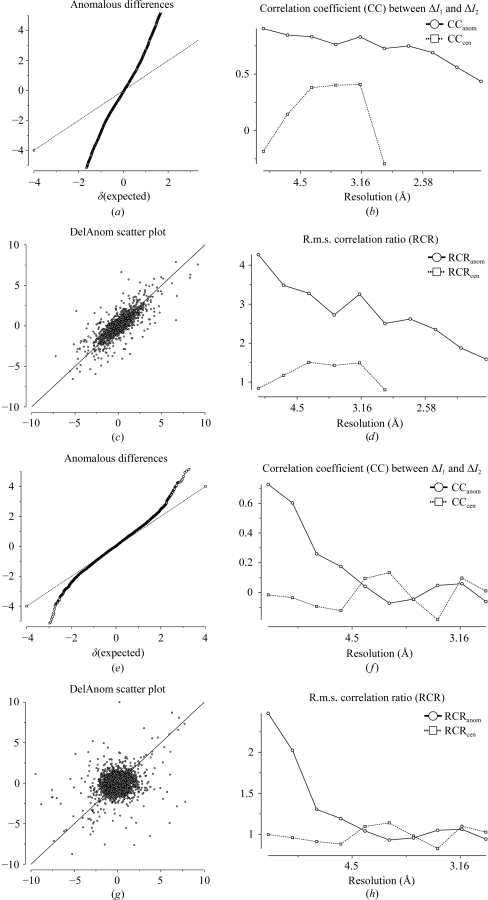
Detection of anomalous signal. (*a*–*d*) An example with a very strong anomalous signal, shown by (*a*) a large slope of the normal probability plot of Δ*I*/σ(Δ*I*) values, (*b*) a large correlation coefficient between two Δ*I* estimates from random half-data sets, (*c*) a scatter plot relating two half-data-set values of Δ*I*/σ(Δ*I*) and (*d*) the r.m.s. correlation ratio derived from the scatter plot. (*e*–*h*) The same plots for an example with a weak but still useful anomalous signal.

A data set contains measurements of reflections from both Bijvoet pairs *I* ^+^(*h k l*) and *I* ^−^(−*h* −*k* −*l*), which will be systematically different if there is anomalous scattering. Fig. 5 ▶ shows some statistics from *SCALA* for a case with a very strong anomalous signal and for one with a weak but still useful signal. Figs. 5 ▶(*a*) and 5 ▶(*e*) show normal probability plots (Howell & Smith, 1992 ▶) of Δ*I*_anom_/σ(Δ*I*_anom_), where Δ*I*_anom_ = *I* ^+^ − *I* ^−^ is the Bijvoet difference: the central slope of this plot will be >1 if the anomalous differences are on average greater than their error. Another way of detecting a significant anomalous signal is to compare the two estimates of Δ*I*_anom_ from random half data sets, Δ*I*_1_ and Δ*I*_2_ (provided there are at least two measurements of each, *i.e.* a multiplicity of roughly 4). Figs. 5 ▶(*b*) and 5 ▶(*f*) show the correlation coefficient between Δ*I*_1_ and Δ*I*_2_ as a function of resolution: Fig. 5 ▶(*f*) shows little statistically significance beyond about 4.5 Å resolution. Figs. 5 ▶(*c*) and 5 ▶(*g*) show scatter plots of Δ*I*_1_ against Δ*I*_2_: this plot is elongated along the diagonal if there is a large anomalous signal and this can be quantitated as the ‘r.m.s. correlation ratio’, which is defined as (root-mean-square deviation along the diagonal)/(root-mean-square deviation perpendicular to the diagonal) and is shown as a function of resolution in Figs. 5 ▶(*d*) and 5 ▶(*h*). The plots against resolution give a suggestion of where the data might be cut for substructure determination, but it is important to note that useful albeit weak phase information extends well beyond the point at which these statistics show a significant signal.

## 5. Estimation of amplitude |*F*| from intensity *I*

If we knew the true intensity *J* we could just take the square root, |*F*| = *J* ^1/2^. However, measured intensities have an error, so a weak intensity may well be measured as negative (*i.e.* below background); indeed, multiple measurements of a true intensity of zero should be equally positive and negative. This is one reason why when possible it is better to use *I* rather than |*F*| in structure determination and refinement. The ‘best’ (most likely) estimate of |*F*| is larger than *I* ^1/2^ for weak intensities, since we know |*F*| > 0, but |*F*| = *I* ^1/2^ is a good estimate for stronger intensities, roughly those with *I* > 3σ(*I*). The programs *TRUNCATE* and its newer version *CTRUNCATE* estimate |*F*| from *I* and σ(*I*) as 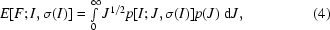
where the prior probability of the true intensity *p*(*J*) is estimated from the average intensity in the same resolution range (French & Wilson, 1978 ▶).

## 6. Intensity statistics and crystal pathologies

At the end stage of data reduction, after scaling and merging, the distribution of intensities and its variation with resolution can indicate problems with the data, notably twinning (see, for example, Lebedev *et al.*, 2006 ▶; Zwart *et al.*, 2008 ▶). The simplest expected intensity statistics as a function of resolution *s* = sinθ/λ arise from assuming that atoms are randomly placed in the unit cell, in which case 〈*I*〉(*s*) = 〈FF*〉(*s*) = 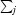 *g*(*j*, *s*)^2^, where *g*(*j*, *s*) is the scattering from the *j*th atom at resolution *s*. This average intensity falls off with resolution mainly because of atomic motions (*B* factors). If all atoms were equal and had equal *B* factors, then 〈*I*〉(*s*) = *C*exp(−2*Bs* ^2^) and the ‘Wilson plot’ of log[〈*I*〉(*s*)] against *s* ^2^ would be a straight line of slope −2*B*. The Wilson plot for proteins shows peaks at ∼10 and 4 Å and a dip at ∼6 Å arising from the distribution of inter­atomic spacings in polypeptides (fewer atoms 6 Å apart than 4 Å apart), but the slope at higher resolution does give an indication of the average *B* factor and an unusual shape can indicate a problem (*e.g.* 〈*I*〉 increasing at the outer limit, spuriously large 〈*I*〉 owing to ice rings *etc*.). For detection of crystal pathologies we are not so interested in resolution dependence, so we can use normalized intensities *Z* = *I*/〈*I*〉(*s*) ≃ |*E*|^2^ which are independent of resolution and should ideally be corrected for anisotropy (as is performed in *CTRUNCATE*). Two useful statistics on *Z* are plotted by *CTRUNCATE*: the moments of *Z* as a function of resolution and its cumulative distribution. While 〈*Z*〉(*s*) = 1.0 by definition, its second moment 〈*Z* ^2^〉(*s*) (equivalent to the fourth moment of *E*) is >1.0 and is larger if the distribution of *Z* is wider. The ideal value of 〈*E* ^4^〉 is 2.0, but it will be smaller for the narrower intensity distribution from a merohedral twin (too few weak reflections), equal to 1.5 for a perfect twin and larger if there are too many weak reflections, *e.g.* from a noncrystallographic translation which leads to a whole class of reflections being weak. The cumulative distribution plot of *N*(*z*), the fraction of reflections with *Z* < *z*, against *z* will show a characteristic sigmoidal shape if there are too few weak reflections in the case of twinning. The most reliable test for twinning seems to be the *L* test (Padilla & Yeates, 2003 ▶), examining *N*(|*L*|), the cumulative value of |*L*|, where *L* = [*I*(**h**_1_) − *I*(**h**_2_)]/[*I*(**h**_1_) + *I*(**h**_2_)] for pairs of reflections **h**_1_ and **h**_2_ close in reciprocal space and unrelated by crystal symmetry. For untwinned data *N*(|*L*|) = |*L*|, giving a diagonal plot, while for twinned data *N*(|*L*|) > |*L*| and *N*(|*L*|) = |*L*|(3 − *L* ^2^)/2 for a perfect twin. This test seems to be largely unaffected by anisotropy or translational non­crystallographic symmetry which may affect tests on *Z*. The calculation of *Z* = *I*/〈*I*〉(*s*) depends on using a suitable value for *I*/〈*I*〉(*s*) and noncrystallographic translations or uncorrected anisotropy lead to the use of an inappropriate value for 〈*I*〉(*s*). These statistical tests are all unweighted, so it may be better to exclude weak high-resolution data or to examine the resolution dependence of, for example, the moments of *Z* (or possibly *L*). It is also worth noting that fewer weak reflections than expected may arise from unresolved closely spaced spots along a long real-space axis, so that weak reflections are contaminated by neighbouring strong reflections, thus mimicking the effect of twinning.

## 7. Summary: questions and decisions

In the process of data reduction, a number of decisions need to be taken either by the programs or by the user. The main questions and con­siderations are as follows.

(i) What is the point group or Laue group? This is usually unambiguous, but pseudosymmetry may confuse the programs and the user. Close examination of the scores for individual symmetry elements from *POINTLESS* may suggest lower symmetry groups to try.
(ii) What is the space group? Distinction between screw axes and pure rotations from axial systematic absences is often unreliable and it is generally a good idea to try all the likely space groups (consistent with the Laue group) in the key structure-solution step: either molecular-replacement searches or substructure searches in experimental phasing. For example, in a primitive orthorhombic system the eight possible groups *P*2_*x*_2_*x*_2_*x*_ should be tried. This has the added advantage of providing some negative controls on the success of the structure solution.
(iii) Is there radiation damage: should data collected after the crystal has had a high dose of radiation be ignored (possibly at the expense of resolution)? Cutting back data from the end may reduce completeness and the optimum trade-off is hard to choose.
(iv) What is the best resolution cutoff? An appropriate choice of resolution cutoff is difficult and sometimes seems to be performed mainly to satisfy referees. On the one hand, cutting back too far risks excluding data that do contain some useful information. On the other hand, extending the resolution further makes all statistics look worse and may in the end degrade maps. The choice is perhaps not as important as is sometimes thought: maps calculated with slightly different resolution cutoffs are almost indistinguishable.
(v) Is there an anomalous signal detectable in the intensity statistics? Note that a weak anomalous signal may still be useful even if it is not detectable in the statistics. The statistics do give a good guide to a suitable resolution limit for location of the substructure, but the whole resolution range should be used in phasing.
(vi) Are the data twinned? Highly twinned data sets can be solved by molecular replacement and refined, but probably not solved, by experimental phasing methods. Partially twinned data sets can often be solved by ignoring the twinning and then refined as a twin.
(vii) Is this data set better or worse than those previously collected? One of the best things to do with a bad data set is to throw it away in favour of a better one. With modern synchrotrons, data collection is so fast that we usually have the freedom to collect data from several equivalent crystals and choose the best.

In most cases the data-reduction process is straightforward, but in difficult cases critical examination of the results may make the difference between solving and not solving the structure.

## Figures and Tables

**Figure 6:**
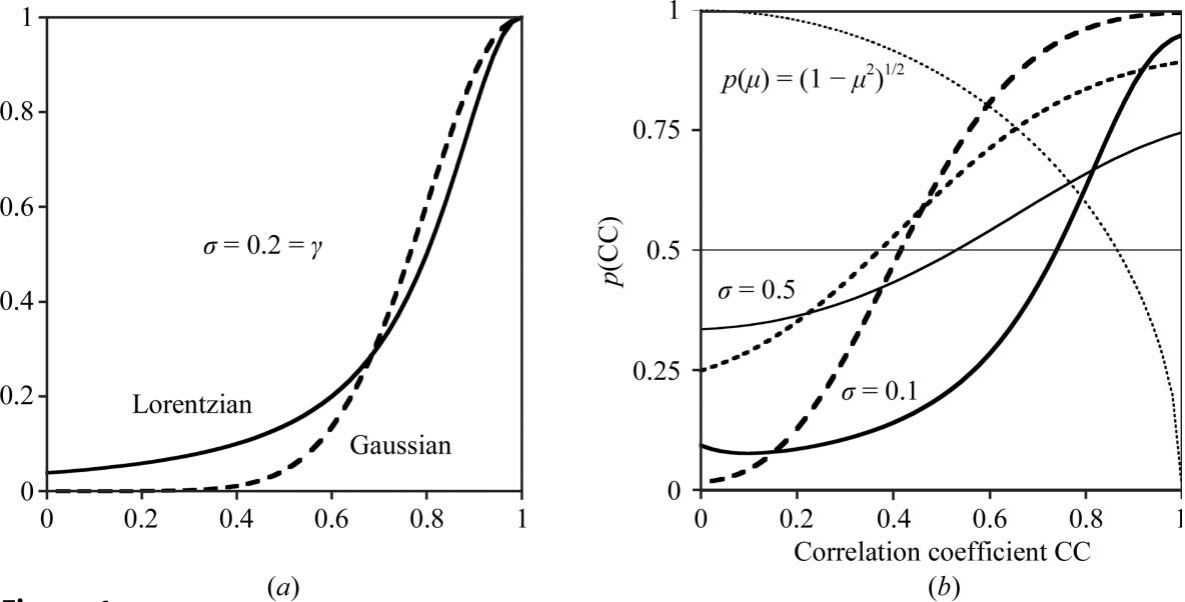
Probability functions for correlation coefficients. (*a*) Comparison of Gaussian (dashed line) and Cauchy–Lorentzian (solid line) distributions with mean 1.0 and width parameter (σ or γ) = 0.2; the Lorentzian distribution has more extensive tails. (*b*) The effect on the modelled distribution *p*(CC) of σ(CC) and including *p*(μ) = (1 − μ^2^)^1/2^ (dotted line). A larger value of σ(CC) broadens the distribution (thin lines, σ = 0.5; thick lines, σ = 0.1). The effect of including the *p*(μ) term (solid lines) is to shift the point at which *p*(CC) rises above 0.5 to a larger value of CC than without it (dashed lines).

**(a):** Scores for each symmetry element. *R*_meas_ = 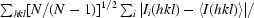 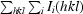; CC is the linear correlation coefficient between normalized intensities *E* ^2^; Z-CC = CC/σ(CC), where σ(CC) is estimated from random uncorrelated observations.

Likelihood	Z-CC	CC	No.	*R*_meas_		Symmetry	Operator
0.948	9.54	0.95	12122	0.097		Identity	
0.942	9.44	0.94	18346	0.121	***	Twofold *l* (001)	{−*h* −*k* +*l*}
0.949	9.58	0.96	30259	0.097	***	Twofold *h* (100)	{+*h* −*k* −*l*}
0.912	9.15	0.92	17427	0.120	***	Twofold *k* (010)	{−*h* +*k* −*l*}

**(b):** Scores for possible subgroups of the lattice group *Pmmm*, giving a clear indication that *Pmmm* is the correct Laue symmetry. CC− is the correlation coefficient for all lattice symmetry elements not present in the Laue group; Zcc− = CC−/σ(CC−); NetZcc = Zcc+ − Zcc−; Likelihood is a probability estimate based on CC and CC− (see Appendix *A* [App appa]); Delta is the angular deviation between the test lattice symmetry and the lattice symmetry implied by the Laue group.

Laue group	Likelihood	NetZcc	Zcc+	Zcc−	CC	CC−	*R*_meas_	*R*−	Delta	Reindex
*Pmmm*	0.985***	9.35	9.35	0.00	0.94	0.00	0.11	0.00	0.0	[*h*, *k*, *l*]
*P*12/*m*1	0.006	0.38	9.56	9.18	0.96	0.92	0.10	0.12	0.0	[−*k*, −*h*, −*l*]
*P*12/*m*1	0.005	−0.01	9.38	9.39	0.94	0.94	0.11	0.11	0.0	[−*h*, −*l*, −*k*]
*P*12/*m*1	0.003	−0.13	9.31	9.44	0.93	0.94	0.11	0.11	0.0	[*h*, *k*, *l*]
*P*−1	0.000	0.22	9.54	9.32	0.95	0.93	0.10	0.11	0.0	[*h*, *k*, *l*]

**(c):** Fourier analysis of axial reflections for systematic absences, indicating the presence of 2_1_ screws along each principal axis. Peak height is the value at 1/2 the cell in Fourier space relative to the origin.

Axis	No.	Peak height	SD	Probability	Condition
Screw axis 2_1_ [**a**]	3	1.000	0.296	0.889**	*h*00: *h* = 2*n*
Screw axis 2_1_ [**b**]	26	1.000	0.142	0.971***	0*k*0: *k* = 2*n*
Screw axis 2_1_ [**c**]	46	0.997	0.097	0.986***	00*l*: *l* = 2*n*

**(d):** Summary of the best solution. The ‘confidence’ scores are derived from the total probability of the best solution *p*_best_ and that for the next best solution *p*_next_: confidence = [*p*_best_(*p*_best_ − *p*_next_)]^1/2^.

Best solution	Space group *P*2_1_2_1_2_1_
Reindex operator	[*h*, *k*, *l*]
Laue-group probability	0.985
Systematic absence probability	0.851
Total probability	0.838
Space-group confidence	0.784
Laue-group confidence	0.982

**Table 2:** Scores for potential individual symmetry operators for a pseudo-cubic example Items are as in Table 1 ▶. The unit-cell parameters are *a* = 79.15, *b* = 81.33, *c* = 81.15 Å, α = β = γ = 90°, *i.e. a* ≃ *b* ≃ *c*. Only the orthorhombic symmetry operators are present (marked ***) and the true space group is *P*2_1_2_1_2_1_.

Likelihood	Z-CC	CC	No.	*R*_meas_		Symmetry	Operator
0.952	9.68	0.97	14733	0.074		Identity	
0.943	9.50	0.95	12928	0.163	***	Twofold *l* (0 0 1)	{−*h*, −*k*, *l*}
0.948	9.59	0.96	12542	0.098	***	Twofold *k* (0 1 0)	{−*h, k*, −*l*}
0.944	9.52	0.95	17039	0.140	***	Twofold *h* (1 0 0)	{*h*, −*k*, −*l*}
0.051	0.55	0.05	13921	0.689		Twofold (1 −1 0)	{−*k*, −*h*, −*l*}
0.057	0.12	0.01	16647	0.734		Twofold (0 1 −1)	{−*h*, −*l*, −*k*}
0.069	2.87	0.29	10540	0.470		Twofold (1 0 −1)	{−*l*, −*k*, −*h*}
0.051	0.62	0.06	12229	0.690		Twofold (1 1 0)	{*k, h*, −*l*}
0.065	2.68	0.27	12829	0.484		Twofold (1 0 1)	{*l*, −*k*, *h*}
0.058	0.10	0.01	17477	0.736		Twofold (0 1 1)	{−*h*, *l*, *k*}
0.059	0.06	0.01	24869	0.824		Threefold (1 −1 −1)	{−*k*, *l*, −*h*} {−*l*, −*h*, *k*}
0.059	0.04	0.00	27024	0.814		Threefold (1 1 −1)	{−*l*, *h*, −*k*} {*k*, −*l*, −*h*}
0.058	0.08	0.01	22508	0.782		Threefold (1 −1 1)	{*l*, −*h*, −*k*} {−*k*, −*l*, *h*}
0.060	0.02	0.00	23818	0.824		Threefold (1 1 1)	{*k*, *l*, *h*} {*l*, *h*, *k*}
0.051	0.58	0.06	25338	0.635		Fourfold *l* (0 0 1)	{−*k*, *h*, *l*} {*k*, −*h*, *l*}
0.062	2.49	0.25	23516	0.476		Fourfold *k* (0 1 0)	{*l*, *k*, −*h*} {−*l*, *k*, *h*}
0.065	−0.15	−0.02	26383	0.739		Fourfold *h* (1 0 0)	{*h*, *l*, −*k*} {*h*, −*l*, *k*}

## Appendix A Scoring schemes for the program *POINTLESS*

*POINTLESS* is a program for scoring the consistency of a set of unmerged diffraction intensities against the possible space groups, given the unit cell and cell centring, in order to identify the most probable space group. It will optionally handle nonchiral space groups, but by default restricts its choices to chiral groups. The scoring schemes used in *POINTLESS* for determination of likely Laue groups and space groups have changed somewhat from those described in Evans (2006 ▶). This appendix outlines the main scoring algorithms used in the current version (1.5.7 at the time of writing). Scoring uses the correlation coefficient CC between normalized intensities *E*_**h**_^2^. Normalization makes 〈*E*_**h**_^2^〉 = 1 over all resolution ranges. The correlation coefficient is less sensitive to the fact that the observations are not on a common scale than are ‘difference’ scores (*i.e.* those involving difference terms, such as *R*_merge_); putting the observations on a common scale would require us to know the symmetry that we are trying to determine. The only correction to the intensities applied prior to scoring is a simple linear time-dependent *B* factor, which is used as a crude radiation-damage correction. It would be an improvement to first perform some rough scaling in Laue group *P*1 to remove gross scaling errors before symmetry determination and this may be performed in the future.

The correlations are used to generate probabilities for the presence and absence of each possible symmetry operation and then combined to give the likelihood of each space group. The space group with the maximum likelihood can then be selected for data merging and structure solution.

### A1. Scoring individual symmetry elements

The first stage of the algorithm implemented in *POINTLESS* is the identification of the highest lattice symmetry compatible with the unit-cell parameters taken from the input file or files, within a tolerance (the current default is 2° on unit-cell angles and an equivalent tolerance on unit-cell lengths). The symmetry information in the file is ignored, except for lattice centring. A list of all rotational symmetry elements is generated for this lattice and they are first scored individually from the correlation coefficient CC on *E* ^2^ between all pairs of observations related by each putative symmetry operator *S*. The likelihood of this crystallographic symmetry element being present is then estimated. To do this, we want to take into account (i) errors in CC, notably that arising from a small number of observation pairs, and (ii) that the expected value of CC if the symmetry element is not present *E*(CC; !*S*) may be greater than 0, and possibly much greater, if pseudo-symmetry is present: for example, CC = 0.6 probably does not indicate that crystallographic symmetry is present.

#### A1.1. Estimation of σ(CC) as a function of sample size

The error in the correlation coefficient CC will be greater if there are only a few pairs of observations. We can estimate the error σ(CC) using reflection pairs **h**_1_ and **h**_2_, choosing pairs which are not related by potential symmetry but are at similar resolutions. From a list of these pairs we can select a number of groups of size *N* for values of *N* of 3 and upwards: typically a large number of these pairs is available, so we have a large number of such groups, and we use *N* up to a maximum of 200 [beyond this point σ(CC) is small and may be set to a suitable minimum value]. For each *N* we calculate the average and r.m.s. CC over all groups 〈CC〉 and σ(CC) = r.m.s(CC). Empirically, σ(CC) is well approximated as linearly proportional to 1/*N* ^1/2^, *i.e.* σ(CC) = CCsigFac/*N* ^1/2^, where the constant CCsigFac is obtained from a linear fit of σ(CC) to 1/*N* ^1/2^.

#### A1.2. Estimation of *E*(CC; *S*)

Because of errors in the data, the expected value of CC if the symmetry element is present *E*(CC; *S*) will be less than the ideal value of 1.0. We have two ways of estimating *E*(CC; *S*).

(i) Given the list of all *E*_**h**_^2^ and σ(*E*_**h**_^2^), it follows from the definitions of CC and variance that *E*(CC; *S*) = var(*E*_**h**_^2^)/{var(*E*_**h**_^2^) + var[σ^2^(*E*_**h**_^2^)]} = ECC_true_ [this expression can be derived by propagating data pairs with errors (*x* + δ*x*, *y* + δ*y*) through the expression for the correlation coefficient].
(ii) Most data sets contain some observation pairs related by Friedel symmetry (−*h*, −*k*, −*l*) or sometimes the identity operator (if more than 180° of data were collected) and CC_identity_ for these also estimates *E*(CC; *S*).

An average of these estimates is used, with somewhat arbitrary weights depending on the number of observation pairs in CC_identity_,

Here, the limits σ ≥ 0.05 and *N* = 200 (which is unrelated to the previous *N* = 200 in §[Sec seca1.1] *A*1.1) are used to avoid extreme weights.

#### A1.3. Estimation of likelihood of each symmetry element

For each symmetry element *k*, we have CC_*k*_ calculated from *N*_*k*_ pairs, with an estimated error σ(CC_*k*_) = min(0.1, CCsigFac/*N*_*k*_^1/2^): here, σ ≥ 0.1 avoids very small values which would arise from large *N*_*k*_. We then want to estimate the likelihood of this symmetry element being present, *p*(*S*_*k*_; CC_*k*_) = *p*(CC_*k*_; *S*_*k*_)/[*p*(CC_*k*_; S_*k*_) + *p*(CC_*k*_; !S_*k*_)]. The denominator here is a normalization factor to ensure that the probabilities sum to 1, since the individual estimates are unnormalized. In modelling these probabilities *p*(CC), Cauchy–Lorentz distributions are used truncated at −1 and +1, since they seem to fit real data better than Gaussian distributions owing to the larger tails of the Lorentzian distribution (Fig. 6 ▶ *a*). The distribution of CC if the symmetry is present *p*(CC; *S*) can be modelled as a truncated Lorentzian centred on CC_true_ with a width parameter γ = σ(CC_*k*_). Modelling the distribution of CC if the symmetry is not present *p*(CC; !*S*) is more complicated, as we need to consider the possibility that the ‘true’ expected CC is >0 owing to noncrystallographic pseudo-symmetry. We can model the unknown expected CC = μ with a probability distribution *p*(μ) which will decline from a high value when CC = 0 to zero when CC = 1. We can then integrate over possible values of μ from 0 to 1 (to integrate out the unknown variable μ),

where *p*(CC; μ) is centred on μ with a width parameter γ = σ(CC_*k*_). Various model distributions for *p*(μ) were tried on a number of examples and *p*(μ) = (1 − μ^2^)^1/2^ seems to work well, even though this implies that there is a high probability of obtaining CC > 0 in the absence of symmetry. The effect of including the *p*(μ) term on *p*(S_*k*_; CC_*k*_) is to raise the value of CC required to conclude that an individual symmetry element is more likely present than not (Fig. 6 ▶ *b*), *i.e.* where *p*(*S*; CC) > 0.5.

### A2. Scoring Laue groups

All possible point groups compatible with the lattice group (subgroups) can be generated from pairs of lattice group symmetry operators and completing the group (including the identity operator). Each subgroup is characterized by a list of symmetry elements *k* which are either present or absent. For each symmetry element we have *p*(CC_*k*_; S_*k*_) and *p*(CC_*k*_; !*S*_*k*_) calculated as above. We can then calculate for each Laue group (point group) *L*_*j*_ a likelihood *p*(*L*_*j*_) = , where *e*_*jk*_ is either *S*_*k*_ or !*S*_*k*_ as appropriate, normalizing the likelihood such that  = 1, assuming that the *L_j_* are independent.

### A3. Scoring systematic absences

To detect screw axes (and glide planes in nonchiral space groups), reflections in relevant zones (axes or planes) are analysed for systematic absences. This is performed by one-dimensional Fourier analysis of *I*′/σ(*I*) along the axes of interest, where *I*′ is corrected approximately for contamination by neighbouring strong reflections by subtracting a small fraction (by default 0.02) of the neighbours (for axial reflections). Then, for example, a 2_1_ screw axis along *c* should give zero intensities for the 00*l* reflections with odd *l*, so the one-dimensional Fourier transform of *I*′/σ(*I*) should have a peak at *x* = 1/2 in Fourier space the same height as the peak at the origin. This characteristic of screw axes arises from Fourier theory, where it can be shown that the Fourier transform along *c** arising from the whole three-dimensional structure is equivalent to the Fourier transform of the one-dimensional projection of the structure onto the *c* axis in real space; thus, when a screw axis is present the projection effectively halves the repeat distance (cell dimension) in real space, which corresponds to a doubling of the spacing of reflections in reciprocal space. Often, there are only a few measurements along an axis, so an estimate of the error in the Fourier value *v*(*x*), σ(*v*), is estimated from the distribution of a series of ‘control’ Fourier transforms using the same axial indices as the observed data but with their *I*′/σ(*I*) values replaced by values from non-axial reflections at similar resolution. We can denote a general rotation or screw axis as *M*_*q*_, where *q* = 0 for a pure rotation and *q* < *M*. In the case of a twofold axis, for example, we need to consider 2_0_ and 2_1_ (*i.e. M* = 2, *q* = 0 or 1). We estimate the probability of *M*_*q*_ using a Lorentzian distribution in a similar way to that used above (§[Sec seca1.3] *A*1.3): the ideal value of *v*(*x*) is *e*_*q*_ (where the origin peak has been normalized to 1). For example, for a 2_1_ screw axis *e*_*q*_ = 1 and for 2_0_ *e*_*q*_ = 0. We can write the deviation of the observed value *v* from the ideal *e*_*q*_ as *d* = |*v* − *e*_*q*_|. *p*[*q*; *d*, σ(*v*)] is then given by a Lorentzian centred on *e*_*q*_ (= 1.0), width parameter γ = σ(*v*), and truncated at 0 and +1. For the probability of a pure twofold rotation, *q* = 0, *e*_*q*_ = 0, *d* = *v*, we want as before to allow for the possibility that the ‘ideal’ value of *v*(1/2) is greater than 0 owing to pseudo-symmetry, *i.e.*

where *p*(*d*) is currently modelled as (1 − *d*)^2^ and *p*(*q*; *d*) is a Lorentzian as above centred on 0. This analysis works for twofolds and threfolds; for fourfolds and sixfolds the analysis is more complicated since we need to consider several non-independent Fourier points at 1/4 and 1/2 for a fourfold and at 1/6, 1/3 and 1/2 for a sixfold. In these cases we can replace the ‘ideal’ values *e*_*q*_ by a vector of ideals **e**_*q*_ and compare this with the observed vector of values **v**, calculating a probability based on the ‘distance’ between these vectors *d* = |**v** − **e**_*q*_|, integrating and truncating at *d*_max_ instead of +1 as above. Finally, we need to normalize the probabilities such that  = 1 for the *i*th axis or zone. For glide planes, which may be present in nonchiral space groups, the procedure is similar, with a Fourier analysis along the glide direction.

### A4. Combining the scores

For the most likely Laue group or groups, all space groups in that Laue group are considered for their compatibility with the possible systematic absences. For example, in the primitive orthorhombic system we have three axes which may be 2_*q*_ axes, *q* = 0 or 1, with eight possible space groups *P*2_*q*1_2_*q*2_2_*q*3_. The systematic absence probability of each space group is given by multiplying the probabilities for the three axes , *i* = 1, 2, 3. This is then combined with the probability of the Laue group from §[Sec seca2] *A*2 to give a total probability for the space group. In some cases there may be no unique solution: (i) there may be missing data, as it is common to miss a whole axis if it is aligned along the rotation spindle, and (ii) some pairs of space groups cannot be distinguished by systematic absences, including enantiomorphic pairs (*e.g. P*3_1_ and *P*3_2_) and the pairs *I*222/*I*2_1_2_1_2_1_ and *I*23/*I*2_1_3 (further ambiguities are possible in nonchiral space groups). If data for an axis are missing then the space group cannot be determined, so only the Laue group is accepted. For indistinguishable pairs the accepted space group is set to one of them; in future versions the ‘status’ of the space-group information will be stored in the MTZ file, *i.e.* whether just the Laue group is known or the full space group or an enantiomorphic pair. A ‘confidence’ score is calculated from the top two distinguishable possibilities as [*p*_best_(*p*_best_ − *p*_next_)]^1/2^ both for the Laue-group score and the total space-group score.

## Appendix B Adjustment of σ(*I*) estimates in *SCALA*

Integration programs such as *MOSFLM* provide an estimate of the error in the intensity σ(*I*) calculated from a combination of several factors including photon counting statistics (Poisson statistics). This is almost always an underestimate of the real error, so after scaling *SCALA* (like other programs) inflates the σ(*I*) estimates so that on average they explain the residual differences between symmetry-related observations. This ‘correction’ is a function of intensity and uses three parameters with different values for fully recorded and summed partial observations and for each ‘run’ of contiguous batch numbers,

The overall multiplier Sdfac at least in part compensates for the error in the ‘gain’ relating detector pixel values to photon counts and the Sdadd term allows for various instrument instabilities which lead to an error proportional to intensity. The SdB term has no obvious physical meaning, but its inclusion seems to improve the fit to real data. If the standard deviations σ(*I*_*hl*_) were correct, then the normalized deviations δ*_hl_* = (*I*_*hl*_ − 〈*I*′*_h_*〉)/[σ^2^(*I_hl_*) − σ^2^ *I*′*_h_*〉]^1/2^ (sometimes denoted χ_*hl*_), where 〈*I*′_*h*_〉 is the mean of all observations of reflection **h** except *I*_*hl*_, should have a mean of 0.0 and a standard deviation of 1.0. *SCALA* adjusts the parameters to try to make r.m.s.(δ) = 1.0 in all intensity bins by minimizing the residual  summed over all intensity bins *j* using a simplex minimization. The optimum weighting scheme for this residual is not clear; at present the weight used is *N*_*j*_^1/2^, where *N*_*j*_ is the number of observations in the *j*th intensity bin. An initial Sdfac value is estimated by normal probability analysis as described in §[Sec seca3] *A*3 of Evans (2006 ▶). Following this correction the plot of r.m.s.(δ) against 〈*I*〉 output by *SCALA* should be flat and ∼1.
